# Emergency-department accesses in home care paediatric patients: Occurrence and risks of use in a six-year retrospective investigation in Northern Italy

**DOI:** 10.1371/journal.pone.0262085

**Published:** 2021-12-31

**Authors:** Sara Campagna, Alberto Borraccino, Gianfranco Politano, Marco Dalmasso, Aldo Ravaglia, Valerio Dimonte, Maria Michela Gianino

**Affiliations:** 1 Department of Public Health and Paediatrics, University of Torino, Torino, Italy; 2 Department of Control and Computer Engineering, Politecnico of Torino, Torino, Italy; 3 Epidemiology Unit, Local Health Unit TO3, Piedmont Region, Italy; 4 Paediatric General Pratictioner, Local Health Unit TO4, Piedmont Region, Italy; Kaohsuing Medical University Hospital, TAIWAN

## Abstract

**Objective:**

To assess the determinants of ED use in paediatric patients enrolled in an Integrated Paediatric Home Care (IPHC) program.

**Methods:**

A retrospective study was conducted using administrative databases on a cohort of patients enrolled in an IPHC program between January 1st, 2012, and December 31st, 2017, in Northern Italy. ED visits that occurred during the IPHC program were considered. Data were collected considering sociodemographic, clinical and organizational variables. A multivariable stepwise logistic regression analysis was performed. The dependent variable to identify possible associations was ED visit.

**Results:**

A total of 463 ED visits occurred in 465 children, with an incidence rate of 1. The risk of ED visits significantly increased among children involved in the IPHC program after hospital discharge (OR 1.94). Additionally, the risk of ED visits increased significantly as the duration of IPHC increased (OR 5.80 between 101 and 200 days, to OR 7.84 between 201 and 300 days, OR 12.54 between 301 and 400 days and OR 18.67 to more than 400 days).

**Conclusion:**

The overall results represent a practical perspective to contribute improving both the service quality of IPHC and reducing low acuity and improper ED use.

## Background

The emphasis on Paediatric Home Care (PHC) services has increased internationally due to the growing number of children who rely on long-term medical needs and have had complex conditions since birth [1], the availability of High‐Technology interventions outside the hospital and scientific advances in the understanding of insurgent complications [2–4]. PHC services can help children and their families by reducing the instability resulting from hospital readmissions [5, 6]. A 2013 systematic review showed that home care services may lead to high parent satisfaction, to improved quality of life of children and their caregivers and to reduced length of stay [7].

In Italy, PHC programs were introduced in 2001. They are defined as a modality of health and social assistance delivered in the patient’s home in a continuous and integrated way by paediatricians and other healthcare professionals (e.g., medical specialists, nurses, therapists, social workers) [8]. The Italian health system guarantees PHC programs, whose provision depends on the different regional regulations. PHC is provided through two organizational models of care according to the level of intensity, complexity and duration of the care intervention: a) programmed home or ambulatory care in which paediatricians care for patients at their home following a schedule and plan hospital visits according to the patient’s chronic condition; b) integrated PHC (IPHC) consisting of different services in response to medium-/high-complexity medical, nursing and/or social health needs [9]. The IPHC can only be activated following a specific request from the hospital doctor and then coordinated by the paediatrician in charge of the patient. The IPHC service is available from 8AM to 8PM on weekdays, and by on-call medical service on weekend.

Regional guidelines [8] require that assistance be offered to children with both acute and chronic illnesses to provide appropriate care with the aim of improving the child and his or her family quality of life, psychological and physical well-being as well as to reduce the costs associated with long and/or repeated hospitalizations. In addition, terminally ill children should be provided with appropriate palliative care interventions in case of need.

Such a comprehensive home care model is assumed to facilitate hospital discharge and ensure continuity of care, avoiding unnecessary hospital visits or readmission and reducing the use of ED. Scholars have investigated whether IPHC reduces hospital readmission rates and length of hospital stay [7, 10, 11] and, to the authors’ knowledge, no studies have yet discussed the impact of IPHC on ED use in a regional extensive paediatric population through the use of available compulsory administrative data. Aim of the study is identifying sociodemographic, clinical and IPHC variables that could be associated to ED use in paediatric patients enrolled in an IPHC program.

## Methods

A retrospective study was conducted on a cohort of patients enrolled in an IPHC program between January 2012 and December 2017 in Piedmont, Italy. The Piedmont region includes approximately 4.4 million residents, 742,000 of whom are aged 0–18 years [12]. As patients may have needed one or more IPHC events during the six years study period, to avoid any potential correlation due to multiple IPHC enrolments, the study was limited to the first IPHC occurrence.

### Ethics

Study data were obtained by accessing different official administrative medical records, linked through the universal anonymous patient identity number (ID). The ID number is a ministerial certified anonymous univocal code centrally assigned before data storage. The non-reversable anonymous code allows data management to accredited institutions without any further authorization. As all administrative ministerial data are made available in a fully anonymized and de-identified manner, an Ethics Committee approval is not required.

### Data source

IPHC program, patient characteristics and ED admissions data were obtained from two databases of the official Italian National Information System: HC Services official registry instituted with the Ministerial Decree of October 2008 [13], and ED regional registry.

Data from the both registries were merged using the universal anonymous patient identity number (ID) within each study year to ensure that ED visits were linked to the appropriate period of IPHC care.

Travel distance was obtained from the Department for Economic Development and Cohesion (DPS) [14] to ED and expressed as the average distance in minutes between the patient’s home and the ED (≤ 5, 6–20, >20 minutes). Sociodemographic variables registered were sex, age, presence of a nonfamily caregiver and time of arrival in ED. Coherently with the WHO Mortality Database [15], age was grouped in five categories (<1; 1–4; 5–9; 10–14; 15–18 years of age). Clinical variables were related to: triage severity code, complaints/symptoms reported at ED admission, prevalent disorder at IPHC enrolment and at ED visit (defined by International Classification of Diseases version 9 codes available in the data source), death in ED. Other collected information was: IPHC proposer (family paediatrician, hospital, other proponent), IPHC duration in days (≤ 100, 101–200, 201–300, 301–400, >400 days), ED applicant (Emergency Medical Service -EMS, patient or family, paediatrician, other), time of arrival in the ED (grouped in four time slots: 6AM - 2PM, 2PM - 8PM, 8PM - 12PM, 12PM - 6AM) and destination after discharge from the ED (admitted to a hospital ward, discharged at home, dead in the ED).

### Data analysis

To identify the role of the determinants under analysis and to check for possible confounding effects, we analysed data both from a descriptive and regression analysis perspective.

All analyses were performed with R [16]. We also used EpiR [17], Complex Heatmap [18], and GLMs (Generalized Linear Models) to build an automated computational pipeline capable of handling all the pre-processing steps and to provide detailed results. The computational pipeline allowed us to assess the risk of ED visits per stratum with GLM and a descriptive analysis to assess overall how the population was distributed across strata.

We performed a multivariable stepwise logistic regression analysis. We set the likelihood of ED visit as the dependent variable. To check for confounders, we adjusted for each of the other independent variables. The risk of ED visit was reported as an odds ratio (OR) with a 95% confidence interval (CI), and the significance level was set at P<0.05. To highlight whether recurring IPHC enrolment introduced any difference because of clustering effects, we computed another stepwise logistic regression analysis. We built the second regression by restricting to the very first IPHC enrolment for each patient. The results showed that the Akaike information criterion (AIC) and Bayesian information criterion (BIC) were not comparable because of the differences in the underlying populations, and we could not resort to analysis of variance (ANOVA) because of different degrees of freedom in the models.

Thus, to assess if any difference was present, we used both fitted models to predict the probability of ED visit while changing the population under examination. For each fitted model, we used both populations (full study sample and first enrolment) to measure the distribution of predicted probabilities.

Furthermore, to correlate the prevalent disorders at IPHC enrolment with those at the ED visit (according to the ICD9 CM) and patients’ age category, we performed a correlation analysis using the Complex Heatmap package [18]. Correlations were reported in percentages and detailed in progressive colour shades to less frequent (lighter shades) to more frequent (darker shades).

## Results

### Patients’ profile

A total of 465 patients were enrolled during the studied period; all age groups were between 0 and 18 years of age (Table 1). Among all the enrolled patients, about 5% of children had a non-family caregiver, the remaining were assisted by a relative. Prevalent disorders at IPHC enrolment were mainly neurological, neoplasm, effects of trauma, perinatal and congenital disorders. Nearly the half of these children (45.4%) were in IPHC for less than 100 days (3 months), and approximately one-third (29.7%) were followed for at least a year (> than 300 days). Most of the IPHC cases were activated by the paediatrician (57%) and the remaining after a hospital or a long-term care facility discharge. Nearly all the patients (98%) lived within 20 minutes from the ED.

**Table 1:** Sociodemographic and clinical characteristics of the patients (n = 465) enrolled in the IPHC program between 2012 and 2017.

Sociodemographic and clinical characteristics	N.	%
**Sex (%)**		
Female	228	49.0
Male	237	51.0
**Age (%)**		
< 1 year old	51	22.0
1–4 years	101	21.7
5–9 years	73	15.7
10–14 years	88	18.9
15–18 years	152	32.7
**Presence of a non-family caregiver**		
Yes	24	5.2
No	441	94.8
Prevalent disorder at IPHC enrolment (ICD-9*)		
Neurological disorder	100	21.5
Neoplasms	75	16.1
Effects of Trauma	52	11.2
Perinatal and congenital disorder	44	9.5
Respiratory disorder	32	6.9
Musculoskeletal and connective disorder	32	6.9
Cardiocirculatory disorder	22	4.7
Urogenital disorder	20	4.3
Endocrine and metabolic disorder	16	3.4
Digestive system disorder	13	2.8
Mental and behavioural problem	8	1.7
Haematological disorder	6	1.3
Infectious disorder	2	0.4
Other disorders	22	4.7
Missing	21	4.5
**Duration of IPHC**		
≤ 100 days	211	45.4
101–200 days	75	16.1
201–300 days	41	8.8
301–400 days	98	21.1
>400 days	40	8.6
**IPHC proposer**		
Family paediatrician	264	56.8
Hospital	118	25.4
Long-term care facilities	83	17.8
**Travel distance to ED in minutes**		
> 5 minutes	164	35.3
6 to 20 minutes	293	63.0
> 20 minutes	8	1.7

IPHC–Integrated Paediatric Home Care; ICD9 –International Classification of Diseases version 9; ED–Emergency department.

### ED visits profile and incidence ratio

Once in the ED (Table 2), 40.4% of the accesses were reported having a medium or a high level of emergency. Prevalent complaints/symptoms at ED arrival were recorded as non-specific symptoms (39%), dyspnoea (8%) and fever (7.7%). In more than 70% of the 463 visits, the family took the patient directly to the ED, whereas the 2.2% were referred by the family paediatricians. EMS was activated in the 18.4% of the cases. The 79% of ED accesses occurred between 8AM and 8PM, approximately two thirds (64%) of visits were discharged home and 36% had a hospital admission.

**Table 2:** Main characteristics at ED arrival and discharge (n = 463 events) among patients enrolled in the IPHC program between 2012 and 2017.

	N.	%
**ED triage codes**		
Low level of emergency	276	59.6
Medium level of emergency	168	36.3
High level of emergency	19	4.1
**Complaints/symptoms reported at ED**		
Non-specific symptoms	287	62.0
Dyspnoea	59	12.7
Fever	56	12.1
Injury	30	6.5
Abdominal pain	13	2.8
Neurological symptoms	12	2.6
Bleeding (not traumatic)	2	0.4
Gynaecological symptoms	2	0.4
Allergic reaction	1	0.2
Cardiac rhythm alteration	1	0.2
**ED applicant**		
Emergency Medical Service	85	18.4
Patient’s family	337	72.8
Family paediatrician	10	2.2
Other applicants	31	6.7
**Time of arrival in ED**		
6 am to 2 pm	223	48.2
2 pm to 8 pm	143	30.9
8 pm to 12 pm	43	9.3
12 pm to 6 am	54	11.7
**Destination after discharge from ED (%)**		
Admitted to hospital	167	36.1
Discharged home	295	63.7
Dead in ED	1	0.2

ED–Emergency department.

During the six-year study period, a total of 463 ED visits occurred (Table 3). The incidence ratio of ED visits by age group decreased as age increased, ranging from over 1.9 (in 1 to 4 years old children) to 0.5 (in 15 to 18 years old ones). The incidence ratio of ED visits linearly increased with the length of IPHC (from 0.3 in fewer than 100 days to 2.6 in more than 400 days). Patients coming from a hospital or a long-term facility had a higher incidence ratio that those referred by a family paediatrician (1.2 and 1.3 vs 0.8 respectively).

**Table 3:** ED visits (N = 463), incidence ratio, adjusted odds ratios (ORs) and 95% confidence intervals (95% CIs) predicted ED use among patients enrolled in the IPHC program between 2012 and 2017.

	ED visits		Incidence ratio	Adj OR‡	[95% CI]
	N	%			
**Sex**					
Male	207	44.7	0.9	1	
Female	256	55.3	1.1	1.42	[0.94–2.14]
**Age**					
< 1 year old	87	18.8	1.7	1	
1–4 years	193	41.7	1.9	1.18	[0.61–2.28]
5–9 years	61	13.2	0.8	0.82	[0.38–1.78]
10–14 years	50	10.8	0.6	0.46*	[0.22–0.95]
15–18 years	72	15.6	0.5	0.62	[0.31–1.25]
**Duration of IPHC in days**					
Fewer than 100 days	54	11.7	0.3	1	
Between 101 and 200 days	67	14.5	0.9	5.80*	[3.23–10.41]
Between 201 and 300 days	52	11.2	1.3	7.84*	[3.91–15.71]
Between 301 and 400 days	187	40.4	1.9	12.54*	[7.29–21.59]
More than 400 days	103	22.2	2.6	18.67*	[9.18–37.95]
**Presence of a nonfamily caregiver**					
Yes	32	6.9	1.3	1	
No	431	93.1	1.0	1.01	[0.44–2.31]
**IPHC proposer**					
Family paediatrician	214	46.2	0.8	1	
Hospital	140	30.2	1.2	1.94*	[1.18–3.19]
Long-term care facilities	109	23.5	1.3	1.89*	[1.10–3.23]
**Travel distance to ED in minutes**					
≥ 5 minutes	135	29.2	0.8	1	
6 to 20 minutes	316	68.3	1.1	1.00	[0.65–1.56]
> 20 minutes	2	0.4	0.3	0.81	[0.12–5.52]
Missing	10	2.2			
**Prevalent disorder at IPHC enrolment (ICD-9)**					
Other disorders	25	5.4	1.1	1	
Haematological disorder	15	3.2	2.5	6.74	[0.62–73.1]
Mental and behavioural problem	15	3.2	1.9	1.40	[0.28–6.97]
Perinatal and congenital disorder	80	17.3	1.8	1.26	[0.45–3.52]
Neurological disorder	125	27.0	1.3	1.27	[0.51–3.16]
Urogenital disorder	25	5.4	1.3	1.30	[0.38–4.40]
Endocrine and metabolic disorder	15	3.2	0.9	0.42	[0.12–1.52]
Respiratory disorder	28	6.0	0.9	0.47	[0.16–1.39]
Neoplasms	51	11.0	0.7	0.58	[0.22–1.50]
Cardiocirculatory disorder	15	3.2	0.7	0.65	[0.20–2.09]
Digestive system disorder	9	1.9	0.7	0.87	[0.20–3.78]
Effects of Trauma	17	3.7	0.3	0.37	[0.13–1.09]
Musculoskeletal and connective disorder	8	1.7	0.3	0.39	[0.12–1.33]
Missing	35	7.6	1.7	1.78	[0.53–5.93]

IPHC–Integrated Paediatric Home Care; ED–Emergency department; ICD9 –International Classification of Diseases version 9; OR–Odds Ratio; ‡ OR of one or more ED visits, computed by logistic regression analysis. All ORs were mutually adjusted for any independent variable; * Statistically significant results with p < 0.05.

Neurologic disorders, perinatal complications and neoplasms accounted for approximately 50% of the ED visits, whereas incidence ratios were higher among haematological (2.5), mental and behavioural disorders (1.9), and perinatal and congenital disorders (1.8).

**Fig 1:**
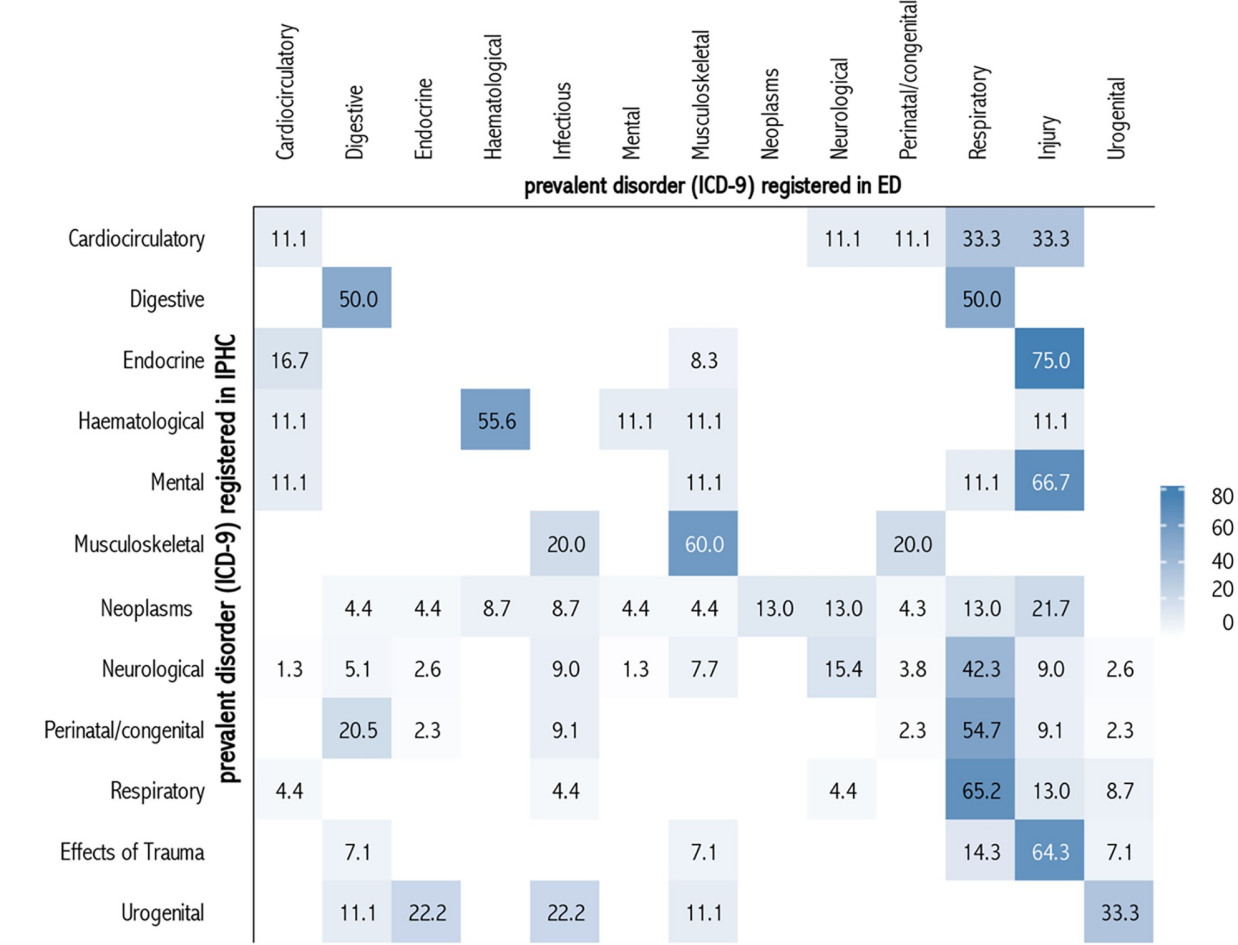
Heatmap matrix of the associations (%) between prevalent disorders (ICD- 9) registered in integrated IPHC and in ED between 2012 and 2017.

**Fig 2:**
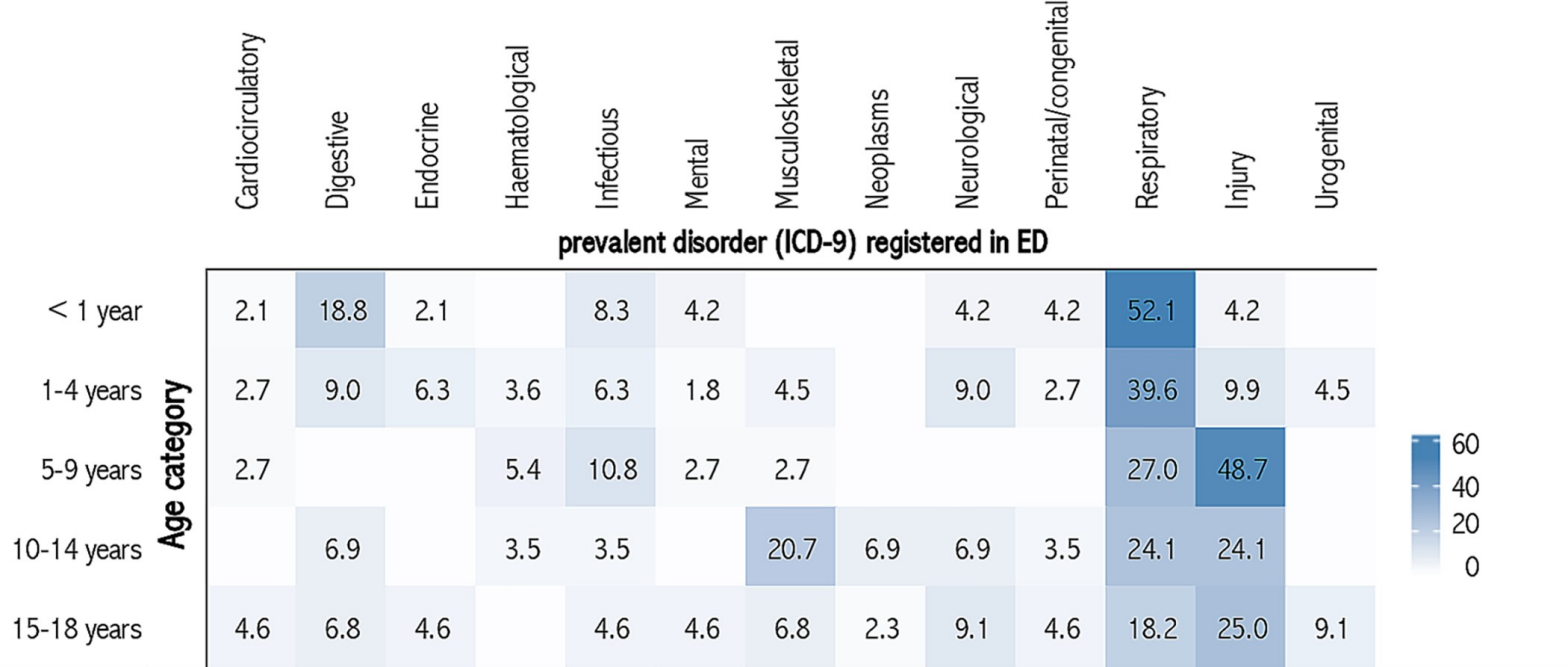
Heatmap matrix of the associations (%) between patient’s age category and prevalent disorders (ICD- 9) registered in ED between 2012 and 2017.

Overall, ED visits were determined by the underlying prevalent disorder at the IPHC enrolment. The most frequent conditions requiring an ED visit were respiratory diseases and acute trauma in previous mental and endocrinological disorders or effect of trauma (Fig 1). As for the age of the patient (Fig 2), respiratory diseases were the most represented conditions for an ED visit, in particular among the youngest (52.1%), whereas trauma involved mostly 5 to 9 years old children (48.7%).

### Determinants of ED use

The regression analysis reported in Table 3, showed an increased significant risk of ED visits among children involved in the IPHC program after hospital or residential facility discharge (OR 1.94 95% CI 1.18, 3.19 and OR 1.89 95% CI 1.10, 3.23, respectively), and as the length of the IPHC increased (OR 5.80 95% CI 3.23–10.41, between 101 and 200 days; OR 7.84 95% CI 3.91–15.71, between 201 and 300 days; OR 12.54 95% CI 7.29–21.59, between 301 and 400 days; OR 18.67 95% CI 9.18–37.95 to more than 400 days).

Social demographic factors, such as sex, presence of non-family caregivers, distance to the ED, and clinical factors did not seem to be associated with the risk of ED visit. As for the age, only children between 10 to 14 years old showed a reduction on the risk of ED access when compared to the youngest (OR 0.46 95% CI 0.22–0.95).

## Discussion

Consequences of neurological, perinatal or congenital disorders were the most prevalent conditions requiring a HC program and IPHC was principally activated by the family paediatrician. Children discharged from a hospital or a long-term care facility showed the highest likelihood of having an ED access. Among all ED visits, more than the 60% were home discharged. The prevalent complaints reported in ED were mainly registered as non-specific symptoms underlying the difficulty of dealing with complex medical conditions for health professionals and families.

Although indeed appreciable, medical progress that allowed children with severe conditions to survive was not coupled with an adequate improvement in home care services, leaving an increased number of parents alone in caring their children [19]. Even when the family paediatrician or the home care team was available, most ED visits occurred during the daytime under the decision and responsibility of the families and by means of EMS.

Evidence suggests that parents’ ability to differentiate urgent from non-urgent conditions is mainly affected by psychosocial factors, such as the parents’ level of anxiety for their child’s condition, trust in the hospital rather than the primary care and the level of satisfaction with home care services [20–22].

Moreover, insufficient parent education was further reported being among the most common causes for an improper ED access [23, 24]. Educational interventions aimed at improving parents’ basic knowledge can contribute to fill the gap in both the competence and the confidence needed to understand the clinical severity. Especially in complex care needs, such interventions proved to be helpful in reducing non-urgent or improper ED visits by helping parents in treating the child condition at home [25, 26]. Studies have further identified that priorities for safety practices at home, such as the training of family care givers, the provision and expertise of services in the community, and the availability and reliability of equipment are handovers between hospitals and community services [27–29]. During IPHC, the common procedures needed by patients often include blood sample collection, enteral feeding and intravenous medication administration, which have clear benefits when performed at home. They need skillful management because they have significant safety risks [30].

In the Piedmont region the IPHC team has no specific paediatric nurse staff and many procedures are unusual to the team engaged at patients’ homes, so children need referral. Working on the quality of IPHC providers through the introduction of quality requirements for home care services and the recruitment of staffing with specific community-centred and paediatric educational requirements are an emerging need of our regional IPHC system and could also improve patients’ health outcomes.

IPHC duration and the likelihood of requiring an ED visit were shown to be interdependent. Both the numbers and the demand for home health care services are indeed increasing. As also evidenced in the adult population [31], the recent technological advances in medical care had both allowed children with complex medical problems to have a higher life expectancy, and increased health services demand. Although more high-quality prospective studies are needed, the use of IPHC duration could represent an indirect measure of the complexity of care.

Beyond their academic relevance, the results of this study could represent an important piece of information for home care administrators and policy makers to foster any control programs aimed at monitoring the health conditions of the paediatric population. To bridge current workforce gaps and to address the fundamental vulnerabilities of the existing systems, a combination of improved opportunities for paediatric home health care training through partnerships with hospital systems can represent a feasible opportunity. Moreover, investing in telehealth with paediatric professionals may also support local nurses or family caregivers without specific experience to safely provide the needed care directly at patients’ home [19, 32, 33].

To provide a broader understanding of our results, some weaknesses must also be taken into consideration. Major limitations are those related to the source of information used, common to all administrative database studies. Administrative data constitute a great advantage for epidemiological studies, but they also suffer problems of reliability and quality. In particular socioeconomic and clinical information are often not correctly filled and outcomes are only collected by using the ICD9 coding system. Moreover, the use of different coding criteria by individuals and institutions could also affect data accuracy [34]. Hence, these shortcomings hampered the study of the effects associated with either clinical, socioeconomic factors or subgroup analyses. Finally, results cannot be generalized to other contexts because they are affected by policies of local organization. HC services by IPHC should have a 24-hour coverage with a higher community-based support. Therefore, transferability to other realities should be carefully assessed.

## Conclusions

The study addressed an issue that is common to many public health systems called to provide continuity of care, combining information that were not initially planned to be used for such a purpose. The supported evidence may offer home care providers and policy makers a practical perspective to improve both the organizational and the quality level of IPHC services and to address low acuity and improper ED use. The study showed that to be safely cared at home, there is the need to foster the team service competences and to improve the system ability to support families in caring their children, when in the presence of complex care needs. To increase knowledge in this field, more high-quality longitudinal studies must be conducted to better investigate both the effectiveness of the service provided and the impact of those actions that guarantee children to receive needed care at home, reducing avoidable ED visits.

## Supporting information

S1 Dataset Absolute frequency of prevalent disorders (ICD- 9) registered in integrated IPHC and absolute frequency of prevalent disorders registered in ED. Period 2012 to 2017. Piedmont Region. Fig 1 dataset.
(XLSX)

S2 Dataset Absolute frequency of prevalent disorders (ICD- 9) registered in integrated IPHC and absolute frequency in patient’s age categories. Period 2012 to 2017. Piedmont Region. Fig 2 dataset.
(XLSX)

## Abbreviations

IPHC: Integrated Paediatric Home Care-services
ED: Emergency Department
OR: Odds Ratio
EMS: Emergency Medicine Service
